# Enhancement of skin rejuvenation and hair growth through novel near-infrared light emitting diode (nNIR) lighting: in vitro and in vivo study

**DOI:** 10.1007/s10103-024-04044-9

**Published:** 2024-04-17

**Authors:** Keonwoo Choi, Hongbin Kim, Sun-young Nam, Chan Yeong Heo

**Affiliations:** 1 Department of Plastic and Reconstructive Surgery, Seoul National University Bundang Hospital, Seongnam, Republic of Korea; 2 Korean Institute of Nonclinical Study, Seongnam, Republic of Korea; 3 H&BIO Corporation/R&D Center, Seongnam, Republic of Korea; 4 Department of Plastic and Reconstructive Surgery, College of Medicine, Seoul National University, Seoul, Republic of Korea

**Keywords:** Light emitting diode (LED), Cell culture, Hair growth, nNIR skin rejuvenation, Hair growth

## Abstract

The study aimed to explore the impact of a novel near-infrared LED (nNIR) with an extended spectrum on skin enhancement and hair growth. Various LED sources, including White and nNIRs, were compared across multiple parameters: cytotoxicity, adenosine triphosphate (ATP) synthesis, reactive oxygen species (ROS) reduction, skin thickness, collagen synthesis, collagenase expression, and hair follicle growth. Experiments were conducted on human skin cells and animal models. Cytotoxicity, ATP synthesis, and ROS reduction were evaluated in human skin cells exposed to nNIRs and Whites. LED irradiation effects were also studied on a UV-induced photoaging mouse model, analyzing skin thickness, collagen synthesis, and collagenase expression. Hair growth promotion was examined as well. Results revealed both White and nNIR were non-cytotoxic to human skin cells. nNIR enhanced ATP and collagen synthesis while reducing ROS levels, outperforming the commonly used 2chip LEDs. In the UV-induced photoaging mouse model, nNIR irradiation led to reduced skin thickness, increased collagen synthesis, and lowered collagenase expression. Additionally, nNIR irradiation stimulated hair growth, augmented skin thickness, and increased hair follicle count. In conclusion, the study highlighted positive effects of White and nNIR irradiation on skin and hair growth. However, nNIR exhibited superior outcomes compared to White. Its advancements in ATP content, collagen synthesis, collagenase inhibition, and hair growth promotion imply increased ATP synthesis activity. These findings underscore nNIR therapy’s potential as an innovative and effective approach for enhancing skin and promoting hair growth.

**Supplementary Information:**

The online version contains supplementary material available at 10.1007/s10103-024-04044-9.

## Introduction

Skin aging is a multifactorial biological process that manifests macroscopically as alterations in appearance owing to the gradual decline in physiological functions [1]. Increase in wrinkles, decrease in elasticity, and marked dryness and skin thinning accompanied by pruritus are the most common symptoms of aging. Histologically, aged skin involves alterations in the structural components of connective tissue, including dermal collagen fibers, solar elastosis, and the deposition of dystrophic elastotic materials [2]. As age increases, reactive oxygen species (ROS) levels increase and mitochondrial function decreases, thereby decreasing adenosine triphosphate (ATP) production. Increased intracellular ROS characterizes aged mesenchymal stem cells and is a factor influencing metabolic dysfunction and skin aging, which frequently occur in the elderly [3]. UV radiation is a powerful external mediator of age-related changes in the skin and is known as “photoaging.” Photoaging accounts for approximately 80% of facial aging [4]. Based on their distinct physical properties, this suggests dermal bond damage, including increased epidermal thickness, irregular pigmentation, typical solar elastosis, looseness, dullness, roughness, and vascular system deformation [5]. The modifications that occur during photoaging are related to structural components, namely collagen elastin and glycosaminoglycans. In older skin, collagen 1 production is low and collagen degradation and elastin degeneration induced by an increase in matrix metalloproteinases (MMPs) are well-known features of photoaging [6]. It induces biological responses, including abnormal ROS accumulation and DNA damage in both epidermal and dermal compartments [7]. UV inhibits ATP production, causing an energy crisis and interfering with optimal skin immunity and DNA repair, but enhanced ATP production can protect against UV immunosuppression, enhance DNA repair, and reduce human skin cancer [8]. It is also well known that acute UV-induced stress induces a local inflammatory state of the skin [9]. Human skin keratinocytes irradiated with UV show increased expression of inflammatory cytokines IL-1β, IL-6, IL-8 and TNF-α through the NF-κB pathway [10].

Since the 1960s, studies on the biological effects of light have been reported [11]. In the 1990s, NASA confirmed the skin treatment effects of Near-Infrared (NIR) light, leading to the commercialization of skin regeneration devices utilizing infrared radiation [12]. This effect became widely known as Low-Level Laser Therapy (LLLT) or Photobiomodulation (PBM). Recently developed LLLT and PBM, which are performed using red NIR wavelengths, are known to have effects such as wound healing [9, 13, 14], pain relief [15], inflammation reduction [16, 17], animal life extension [18], vision improvement [19], cognitive ability improvement [20], and hair loss improvement [21]. Light-emitting diodes (LEDs) are suitable for laser therapy because they emit low levels of power that do not irritate or burn the skin. Recently, an increasing number of reports have shown that red or near-infrared (NIR) laser light stimulates cell activity to promote tissue repair and regeneration. In addition, studies have shown that Near IR wavelengths are absorbed by cytochrome c oxidase, a complex protein of the mitochondria in cells, promoting production of ATP, which is known as the minimal energy unit of human activity [22] .

Visible light, which is the wavelength band of light used in low-power lasers and LED devices, is easy to think of as having no function but can play an important role. In particular, red light-based visible light can penetrate the dermis layer with a longer wavelength band; therefore, it can stimulate resting hair follicles in the dermis layer from the fat layer to facilitate hair follicle growth [23]. Recently, LLLT was evaluated in numerous clinical studies for stimulating hair growth, in which 655 nm red light was found to be the most effective and practical for stimulating hair growth [24].

In this study, we have developed a new Near-Infrared LED (nNIR) that offers a broader spectrum compared to conventional narrow-bandwidth NIR chip and to investigate the potential of nNIR in promoting cell growth, ATP generation, and reducing ROS through cell experiments. To achieve this, we conducted a comparative in vitro analysis against in the wavelength range from blue to NIR. Furthermore, to gain deeper insights into the efficacy of the nNIR for skin rejuvenation and hair loss prevention relative to the commonly used White sources in everyday life, comprehensive cell and in vivo were performed. The findings of this research shed light on the promising applications of the nNIR as a potentially superior alternative for various biomedical and cosmetic purposes.

## Materials and methods

### Cell culture of HaCaT keratinocytes and Hs68 dermal fibroblast cell line

In vitro studies were performed using the human keratinocyte cell line (HaCaT) and human dermal fibroblast cell line. Cells were purchased from the American Type Culture Collection (ATCC). They were cultured in Dulbecco’s modified Eagle’s medium (DMEM; Gibco, Grand Island, NY, USA) containing 10% fetal bovine serum (FBS; Gibco) and 1% penicillin/streptomycin (Gibco, Grand Island, NY, USA) at 37 °C in a 5% CO_2_ incubator. The cells were irradiated using a UVB cross-linker. Hs68 dermal fibroblasts were seeded in 35-mm tissue culture dishes at a density of 2 × 10^4^ cells per tissue. Lipopolysaccharide (LPS) 10 µg/ml was used as a negative control group in the experiment to determine the anti-inflammatory effect and dexamethasone (DEX) 1 μm was used as a positive control group [25]. Retinoic acid 1% was used as a positive control for collagen (*Col1A1*), collagenase (*MMP1, MMP9* and *MMP13*) the moisturizing (*Has3*) qRT-PCR [26].

### LED exposure in vitro

**Fig. 1:**
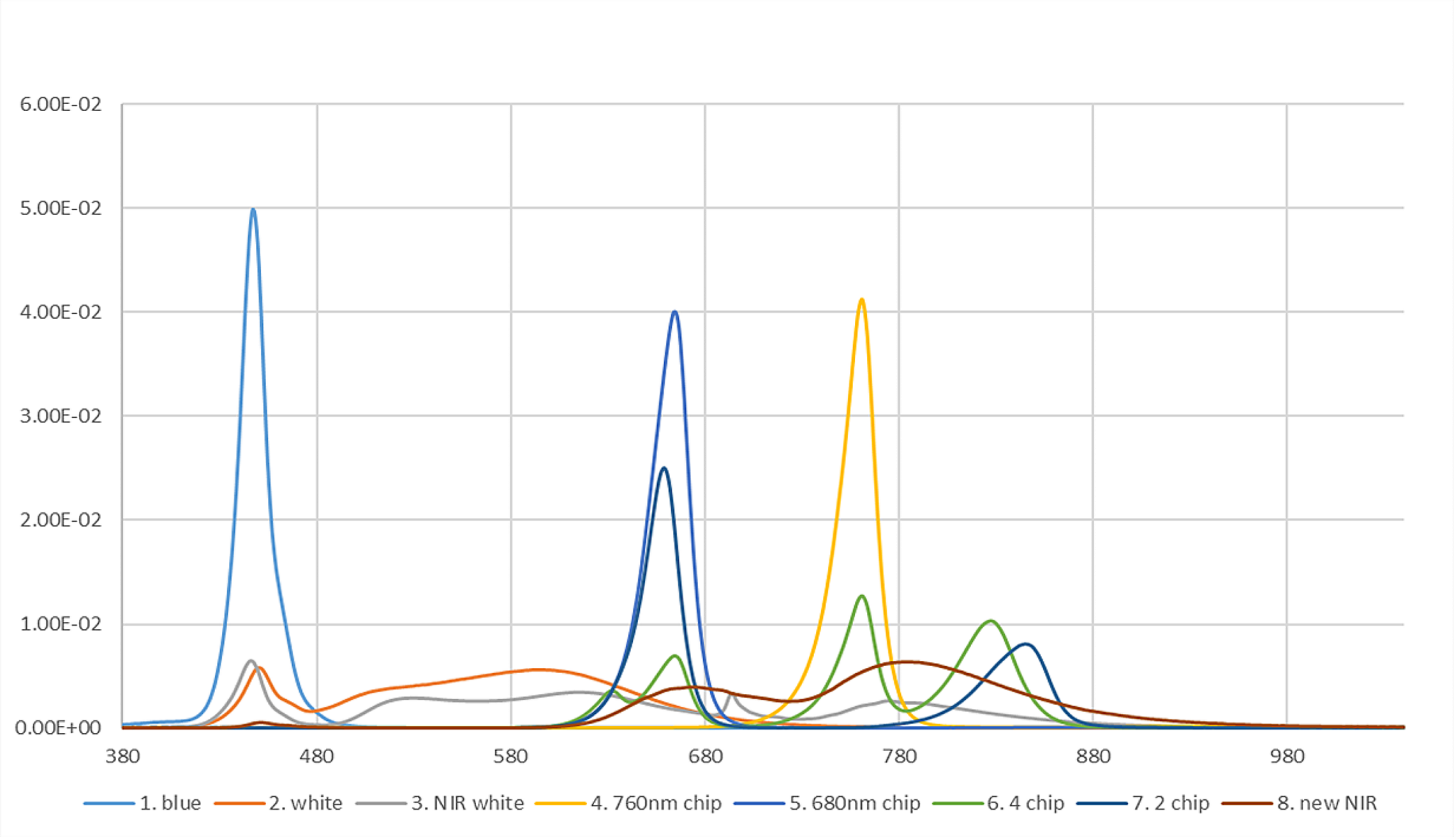
Spectrum profiles of the various LEDs

The Blue, White, NIR white, 680 nm chip, 720 nm chip, 4 chip (620 nm + 680 nm + 760 nm + 830 nm), 2 chip (660 nm + 850 nm) and nNIR irradiator used in this study was manufactured by Samsung Electronics Co., Ltd. The experimental LED illuminating systems, including LED with a white color and nNIR (780 nm centered phosphor), were installed in the cell culture incubator, which was maintained at a temperature of 37 °C and humidified atmosphere of 5% CO_2_. The spectrum for each wavelength is shown in Fig. 1 and Table 1. To reduce light interference, light was irradiated on the surface of the culture plate with partitions, which was 8 cm above the light source. The control group consisted of cells maintained in the dark. The irradiance of the LED sources was 6 mW/cm^2^, as measured using an MK350NPLUS LED meter (UPRtek, Taiwan). To check the effect of doses, we proceeded to each was performed according to the times and intensities indicated in Table 1. Then, cells were washed once with Dulbecco’s phosphate-buffered saline (DPBS, Welgene, Daegu, Republic of Korea) and exposed to 30 mJ/cm2 of UVB using a UV crosslinker (PCL-1000, BoTeck, Gunpo, Republic of Korea) without the culture plate cover.

**Table 1:** Photobiomodulation parameter or LED parameter in vitro

LED parameter	Wavelength (nm)	Power (mW/cm^2^)	Irradiation time (hr)	Energy (J/cm^2^)
Blue	450	0.1	3.5	1.3
			8	2.9
		0.36	1	1.3
			8	10.4
		1.44	2	10.4
			8	41.5
		6	0.5	10.8
			4	86.4
White	380 ∼ 780	6	0.5	10.8
			4	86.4
NIR white	380 ∼ 1040	6	0.5	10.8
			4	86.4
760 nm chip	760	6	0.5	10.8
			4	86.4
680 nm chip	680	6	0.5	10.8
			4	86.4
4chip	620 + 680 + 760 + 830	6	0.5	10.8
			4	86.4
2chip	660 + 850	6	0.5	10.8
			4	86.4
New NIR	580 ∼ 1040	6	0.5	10.8
			4	86.4

### LED exposure of in vivo

Light was irradiated at the top of the cage, which was 20 cm above the light source. The light exposure schedule was 30 min per day, three times a week for 2 weeks. The irradiances of the two light sources were the same at 6 mW/cm^2^ (Table 2).

**Table 2:** Photobiomodulation parameter or LED parameter in vivo

LED parameter	Wavelength (nm)	Power (mW/cm^2^)	Irradiation time (hr)	Energy (J/cm^2^)
White	380 ∼ 780	6	0.5	64.80
New NIR	580 ∼ 1040	6	0.5	64.80

### Cell cytotoxicity assay

Hs68 dermal fibroblasts and HaCaT keratinocytes were seeded in a 96-well plate at a density of 1 × 10^4^ cells per well and cultured for 24 h. After washing with phosphate buffered saline (PBS), the cells were cultured for 24 h in medium containing fetal bovine serum (FBS). After removing the medium, cells were cultured in fresh medium without FBS for 24 h, and White and nNIR were irradiated under irradiation conditions of 10.80 J, 43.20 J, and 86.40 J. MTT (3-[4,5-dimethylthiazol-2-yl]-2,5-diphenyl-tetrazolium bromide; 0.5%) solution was added to the cultured 96-well plate. After incubation for 4 h, the culture medium was removed and 100 µL of dimethyl sulfoxide (DMSO) solution was added to dissolve MTT formazan. Then, absorbance was measured at 570 nm using a microplate spectrophotometer (BioTek, Winooski, VT, USA).

### ROS measurement in Hs68 human dermal fibroblasts

Hs68 cells were seeded in a 96-well plate at a density of 1 × 10^4^ cells per well and cultured for 24 h. After washing with PBS, cells were cultured for 24 h in medium containing FBS. The next day, the cells were treated with White and nNIR, which were 10.80 J, 43.20 J, and 86.40 J. After washing with PBS, 100 µL of DCF-DA solution diluted in the medium without phenol red and FBS was added and incubated at 37 °C for 30 min. After washing again with PBS, 100 µL of H_2_O_2_ (900 µM) solution diluted in the medium without phenol red and FBS was added and reacted in an incubator at 37 °C for 30 min. After washing with PBS, the cells were transferred to a 96-well black-bottom plate, and fluorescence intensity was measured using a microplate spectrophotometer.

### ATP assay in vitro and in vivo

Based on the principle of color development, the amount of ATP produced in Hs68 dermal fibroblasts, HaCaT keratinocytes, or mouse serum was measured using an ATP assay by following the instructions of the assay kit (Abcam, Cambridge, UK). After adding ATP assay buffer to the cell pellet or mouse serum and centrifuging at 4 °C and 13,000 rpm for 5 min, the supernatant was collected in a new tube. After adding cold 1 N perchloric acid solution, interfering proteins were removed for 15 min at 4 °C. After centrifugation at 13,000 rpm for 5 min, the supernatant was collected into a new tube. After adding cold 1 N sodium hydroxide solution, the mixture was allowed to react at 4 °C for 5 min. ATP reaction buffer was added after mixing the 96-well plate with the above solution well with standards and samples; light was blocked, and the plate was incubated at room temperature for 30 min. Thereafter, the fluorescence intensity was measured using a microplate spectrophotometer (excitation, 485 nm; emission, 535 nm) of the 96-well plate on which the reaction was completed.

### qRT-PCR

Total RNA was extracted from either cell line or dorsal skin using Trizol reagent (Invitrogen, Carlsbad, CA, USA) following the manufacturer’s instructions. The single-stranded cDNAs were synthesized using the Prime Script 1st strand cDNA Synthesis kit (Thermo Fisher Scientific, Waltham, MA, USA). All cDNA templates were mixed with SYBR Green Master Mix (Bioneer, Seoul, Korea), and qRT-PCR was performed in an ABI 7700HT thermal cycler (Thermo Fisher Scientific, Waltham, MA, USA). The mRNA cycle threshold (Ct) values were normalized against 36B4 for ΔCt in the same sample, and then to the control sample to produce ΔΔCt. Finally, the fold-change (2 ^−ΔΔCt^) was calculated. Primer sequences for each gene are listed in supply Table 3.

**Table 3:** Specific primer sequences of gene

Gene	Primer	Sequence (5’ to 3’)
*36B4*	Forward	5’-TGG GCT CCA AGC AGA TG-3’
	Reverse	5’-GGC TTC GCT GGC TCC CAC-3’
*COL1A1*	Forward	5’-GTG GCC ATC CAG CTG ACC-3’
	Reverse	5’-AGT GGT AGG TGA TGT TCT GGG AG-3’
*MMP-1*	Forward	5’-ACA GCC CAG TAC TTA TTC CCT TTG-3’
	Reverse	5’-GGG CTT GAA GCT GCT TAC GA-3’
*MMP-9*	Forward	5’-CAC TGT CCA CCC CTC AGA GC-3’
	Reverse	5’-GCC ACT TGT CGG CGA TAA GG-3’
*MMP-13*	Forward	5’-AGT TTG CAG AGC GCT ACC TGA GAT-3’
	Reverse	5’-TTT GCC AGT CAC CTC TAA GCC GAA-3’
*IL-6*	Forward	5’-GCA CTG GCA GAA AAC AAC CT-3’
	Reverse	5’-TCA AAC TCC AAA AGA CCA GTG A-3’
*IL-8*	Forward	5’-CTC TTG GCA GCC TTC CTG ATT-3’
	Reverse	5’-ACT CTC AAT CAC TCT CAG TTC T-3’
*TNF-α*	Forward	5’-CCC AGG GAC CTC TCT CTA ATC-3’
	Reverse	5’-ATG GGC TAC AGG CTT GTC ACT-3’
*HAS-3*	Forward	5’-CTT AAG GGT TGC TTG CTT GC-3’
	Reverse	5’-GTT CGT GGG AGA TGA AGG AA-3’

### Photoaging in vivo mouse model

All animal procedures were approved by the Institutional Animal Care and Use Committee (IACUC) of the Seoul National University Bundang Hospital (IACUC No. BA-2205-343-003-02). The experimental animals used in this study were 7-week-old SKH-1 hairless mice purchased from Orient Bio Co., Ltd., and bred at 20–24 °C, 40–60% humidity and 60 dB or less noise in a 12-hour light-dark cycle. The experimental animals were used after a 1-week acclimatization period, and anesthesia was induced using respiratory anesthesia while maintaining the concentration of isoflurane at 3%. When anesthesia was confirmed, UVB irradiation was used to induce photoaging. In the positive control group, 200 µL of retinoic acid (0.83 mM) was applied to the skin of the back area of mice thrice a week for 2 weeks. In the test group, White and NIR LED were irradiated to the skin of the back area thrice a week for 2 weeks, and the total irradiation amount was 64.80 J/cm^2^. Thereafter, visual evaluation of the experimental animals was carried out at the predetermined time points (day 0 and day 14 after UVB irradiation), and euthanasia was performed in a CO_2_ gas chamber 42 days after the start of the experiment. The skin on the back of the experimental animals was biopsied, and the skin tissue was fixed in 10% formalin solution to produce paraffin blocks.

### Western blot analysis and measurement of type I procollagen

Total proteins were extracted from the dorsal skin in RIPA lysis buffer supplemented with complete protease inhibitor cocktail (GenDEPOT, Barker, USA) following the standard protocol (2). The supernatants were collected after centrifugation at 13,000 rpm at 4℃ for 30 min. Protein concentration was determined using a Bradford assay (Bio-rad, Hercules, CA). For western blot analysis, Equal amount of denatured proteins were separated by SDS-PAGE method and transferred to the nitrocellulose membrane. After blocking non-specific binding by incubation with 5% non-fat milk for 1 h, the membrane was incubated with a primary MMP13 antibody (company) overnight at 4℃ and followed by incubation with HRP-conjugated secondary antibody at 37 ℃ for 2 h. The band was visualized using ECL system (DonginBiotech, Seoul, Korea). Using the ImageJ software, we quantitatively evaluated the gray integration values of each band. Type I procollagen was measured according to the manufacturer’s protocol by the type I procollagen kit (Takara, Tokyo, Japan) and evaluated at 450 nm using microplate reader (BioTek, Winooski, VT, USA).

### Hair growth assay in vivo mouse model

All animal procedures were approved by the Institutional Animal Care and Use Committee (IACUC) of the Seoul National University Bundang Hospital (IACUC No. BA-2205-343-002-01). The experimental animals used in this study were 7-week-old C57BL/6NCrlOri mice (Orient Bio Co., Ltd., KR) that were bred at 20–24 °C, 40–60% humidity60 dB or less noise, in a 12-h light-dark cycle. The experimental animals were used after a 1-week acclimatization period, and anesthesia was induced using respiratory anesthesia while maintaining the concentration of isoflurane at 3%. White and nNIR were irradiated three times a week for 3 weeks, and the total irradiation amount was 97.2 J. Visual evaluation was performed at the time points (days 0, 7, 14, and 21 after hair removal), and euthanasia was performed in a CO_2_ gas chamber on day 21 after hair removal. Visual evaluation (photography) and histological evaluation for hair improvement were performed by applying 3% minoxidil or a test light source to mice (C57BL/6NCrlOri, 7-week-old).

### Skin thickness analysis

In this study, the skin tissues of euthanized experimental animals were fixed with a 10% formalin solution, paraffin blocks were prepared, and tissue slides were prepared by sectioning them to a thickness of 3 μm. After hydration, a hematoxylin and eosin (H&E) solution was used for staining [27]. Skin thickness was measured as the distance from the top of the epidermis to the bottom of the dermis using Image J software.

### Anagen induction score

An anagen induction assay was performed as previously described [28]. On performing histological analysis via H&E staining, anagen induction scores were calculated using an assigned arbitrary score (telogen = 1, anagen I-VI = 2–7), and the mean score was compared between the control mouse groups.

### Statistical analysis

Statistical analyses were performed using SPSS software (IBM, Armonk, NY, USA). Normality was verified using the Wilcoxon signed-rank test. Statistical analysis of the variables for parametric values was performed using a paired *t*-test. Statistical significance was set at *p* < 0.05 indicated using *, *p* < 0.01 indicated using **, and *p* < 0.001 indicated using *** (*p* < 0.05, ^**^p < 0.01, ^***^p < 0.001).

## Results

### nNIR does not cause cell cytotoxicity in skin cells

**Fig. 2:**
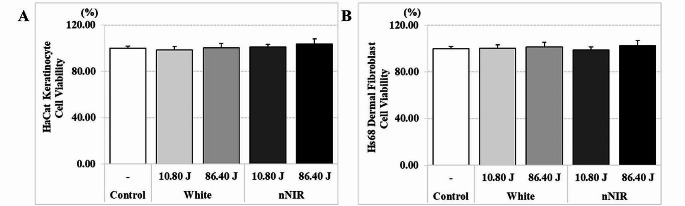
nNIR irradiation does not induce cytotoxicity in Hs68 dermal fibroblasts and HaCaT keratinocytes. (**A**) Cytotoxicity of White and nNIR in HaCaT keratinocytes. (**B**) Cytotoxicity of White and nNIR in Hs68 fibroblasts. Data are mean ± SEM. *N* = 3

We measured cell viability by irradiating skin cells such as HaCaT keratinocytes and Hs68 dermal fibroblasts with White or nNIR to determine whether the nNIR had an effect on cellular toxicity at various doses. As shown in Fig. 2, irradiation of Hs68 fibroblasts and HaCaT keratinocytes with various doses of nNIR or White did not affect cell proliferation. The cells showed a survival rate of more than 90%, and no obvious reduction in cell viability was observed by nNIR or White compared to that of the control group. The results were consistent, except for the cytotoxic on Blue light source (Supply Fig. 1).

### Inhibition of ROS by nNIR in Hs68 dermal fibroblasts

ROS-induced oxidative damage to elastin and collagen in the dermis can cause changes in protein structure, affecting the mechanical properties of the skin [29]. To examine the effect of nNIR or White irradiation on the generation of intracellular ROS, we measured ROS levels in Hs68 dermal fibroblasts (Sup Fig. 1). In the H2O2 treated group, which served as the negative control, ROS levels showed a considerable increase. However, when exposed to White, nNIR, 760 nm chip, and 4 chip at 10.80 J/cm2 and 86.40 J/cm2, ROS levels were significantly decreased compared to the negative control group in Hs68 dermal fibroblast. This result indicates that LED has an antioxidant effect on dermal fibroblasts (Sup Fig. 1).

### Increased in ATP content by nNIR in Hs68 dermal fibroblasts

**Fig. 3:**
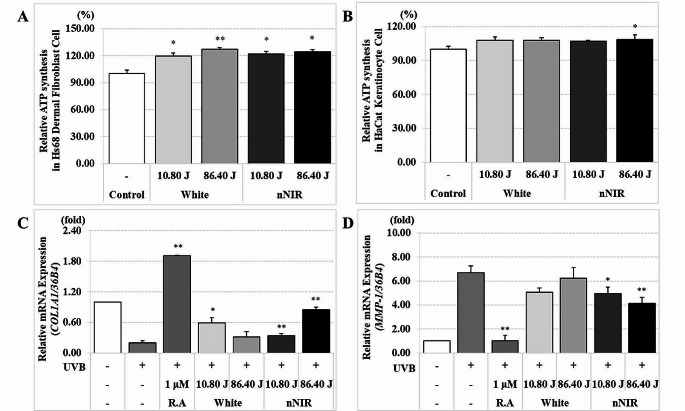
Increase of ATP content and collagen synthesis by nNIR in HaCaT keratinocytes and/or Hs68 dermal fibroblasts. (**A**) The level of ATP content in Hs68 human dermal fibroblasts. (**B**) The level of ATP content in HaCaT keratinoctytes. (**C**) qRT-PCR analysis of *Col1A1* mRNA in HS68 dermal fibroblast. (**D**) qRT-PCR analysis of *MMP1* in HS68 dermal fibroblast cell. Data are mean ± SEM. *N* = 3. *P < 0.05, **P < 0.01

NIR LED wavelengths are absorbed by cytochrome c oxidase, a complex protein of mitochondria in cells, promoting ATP production, known as the minimal energy unit of human activity [30]. We measured the level of ATP content in HaCaT keratinocytes and Hs68 dermal fibroblasts to determine whether the amount of ATP synthesis increased by LED in vitro. The ATP levels were substantially increased in the nNIR irradiated group at 10.80 J/cm^2^, and 86.40 J/cm^2^, and White in Hs68 fibroblasts (Fig. 3). The ATP content markedly increased in the nNIR irradiated group at 86.40 J/cm^2^ compared with that in the control group in HaCaT keratinocytes (Fig. 3). Interestingly, in Hs68 dermal fibroblast, the ATP level was increased by various LEDs, but in HaCat keratinocyte, it was significantly increased only at nNIR, NIR white, and 680 nm at 86.40 J/cm^2^ (Supply Fig. 2).

### nNIR inhibits UVB-induced MMP expression in Hs68 dermal fibroblasts

In skin, UVB-induced activation of the MMP pathway is a characteristic feature of skin aging [4, 8, 31]. We examined the effects of nNIR on UVB-induced expression of *Col1A1* mRNA and collagenase such as *MMP-1*, *MMP-9* and *MMP-13* mRNA using qRT-PCR in Hs68 dermal fibroblasts. A significant reduced in the relative expression level of *MMP1* mRNA was observed in the UVB and nNIR irradiated group compared to that only UVB-induced group both 10.80 J/cm^2^, and 86.40 J/cm^2^ (Fig. 3). The mRNA relative expression level of MMP-9 and MMP-13, another collagenase, was also decreased in UVB and nNIR irradiated group compared to the only UVB-induced group (Supply Fig. 3). Due to the inhibition of collagenase expression, the expression of *COL1A1* mRNA suppressed by UVB increased in the UVB + nNIR irradiated group compared to the UVB only group (Fig. 3). The study confirmed that the broad-spectrum nNIR showed the most significant effects on the main biomarkers for skin wrinkle improvement, *COL1A1* mRNA expression and *MMP-1* mRNA expression. These results suggest the possibility that nNIRs can inhibit UVB-induced collagen damage.

### Anti-inflammatory and moisturizing effect of nNIR on HaCat keratinocyte cell

**Fig. 4:**
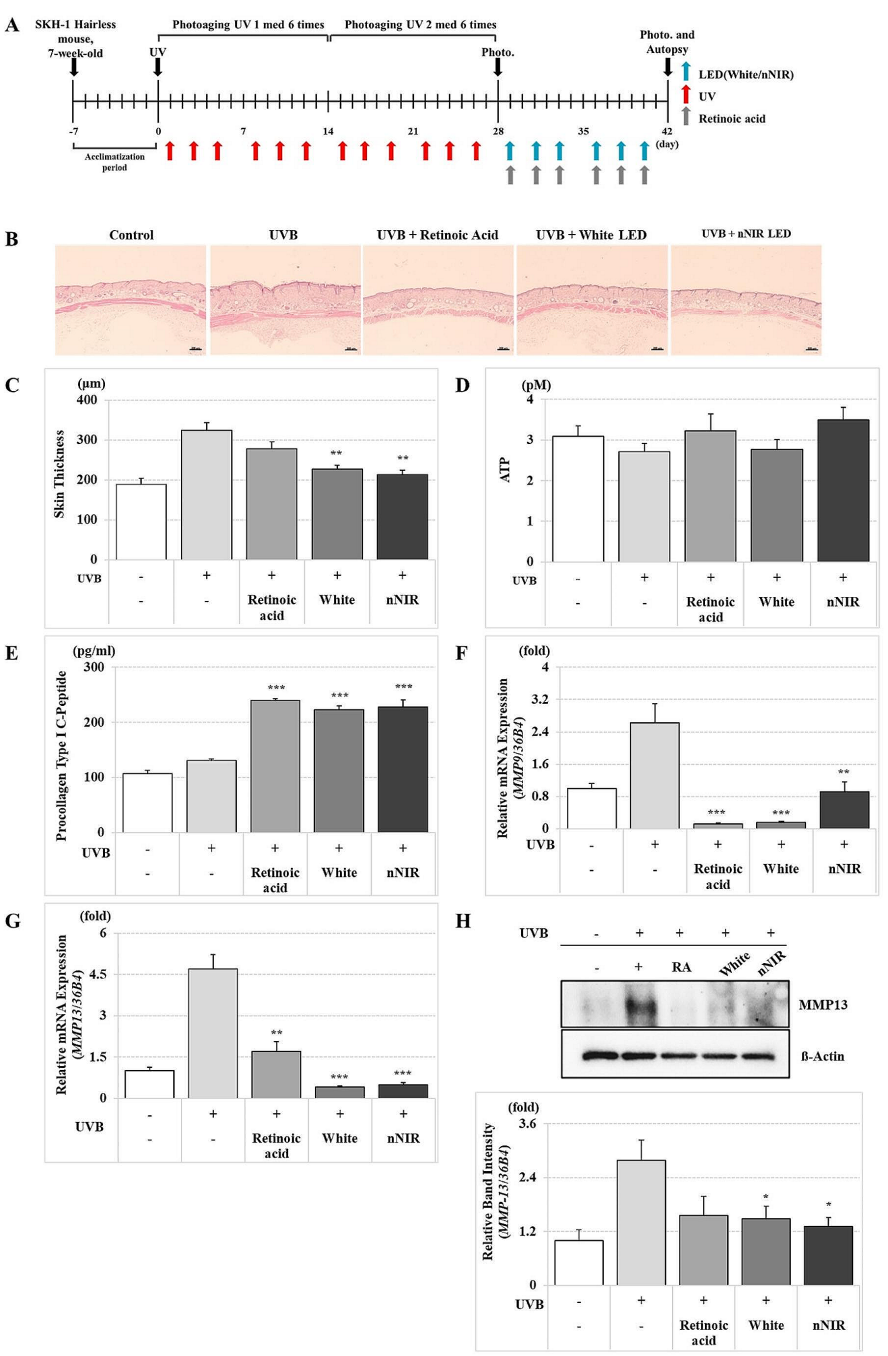
Effects of nNIR on anti-aging in a UV-irradiated photoaging mouse model. (**A**) Schematic presentation of experimental timeline for photoaging model. (**B**) Hematoxylin and eosin (H&E) stain of mice dorsal skin. (**C**) Histological analysis of full mice skin thickness. (**D**) ATP level of mice dorsal skin. **D**) Procollagen type I C-peptide synthesis of mice dorsal skin. **F**) qRT-PCR analysis of *MMP9* in mice dorsal skin. **G**) qRT-PCR analysis of *MMP13* in mice dorsal skin. **H**) Western blot analysis of MMP13 in mice dorsal skin. Data are mean ± SEM. *N* = 7 mice per group. *P < 0.05, **P < 0.01, ***P < 0.001

UVB irradiation potently induces cytokines in the skin such as IL-6, IL-8 and TNF-α [10]. To confirm the anti-inflammatory effect of nNIR, we induced inflammation in HaCaT keratinocyte using LPS. Remarkably, when inflammation was induced with LPS, it was show that the mRNA expression of cytokines such as *IL-6*, *IL-8* and *TNF-α* mRNA increased by 3.78-fold, 3.00-fold and 2.56-fold when only LPS was irradiated, respectively, by nNIR 86.40 J/cm2 irradiation by 1.40-fold, 0.76-fold and 1.14-fold showed a significant decrease, respectively. (Supply Fig. 4). In addition, the *HAS3* mRNA expression, which is known to play an important role in skin moisturizing effect [32], increased by 3.54-fold in the nNIR irradiated group compared to the control group (Supply Fig. 4). These results explain that nNIR is effective in skin improvement such as anti-inflammatory and moisturizing enhancement of skin in vitro.

### Anti-aging effect of nNIR in UV-radiated photoaging in vivo mouse model

To investigate whether nNIR could prevent skin aging, skin photoaging was induced by UVB irradiation on the backs of SKH-1 hairless mice. As epidermal thickening is a major biomarker of photoaging [40], we evaluated the effect of nNIR on UVB-induced skin thickening (Fig. 4). The skin thickness in the negative control (UVB+) group increased by 58.34% compared to that in the control (UVB-) group. As compared to the skin thickness of the negative control (UVB+) based on 100%, it substantially decreased by 33.96% after NIR LED irradiation and by 29.91% after White irradiation in UV-irradiated mouse skin (Fig. 4).

To investigate whether nNIR could increase ATP synthesis in vivo, we measured ATP levels in mouse serum as well as cells. The ATP content decreased by 12.00% for the control (UVB-) as compared to that of the negative control group (UVB+). As compared to the amount of ATP synthesis of the negative control (UVB+) group, the ATP synthesis increased significantly by 28.64% after nNIR irradiation and by 2.01% after White irradiation (*p* < 0.05). The ATP content in the nNIR irradiated group increased by 26.11% compared to that of the White irradiated group (Fig. 4).

We evaluated collagen production after nNIR or White irradiation during UV-induced skin photoaging in vivo and in vitro. As compared to the amount of procollagen production in the negative control group (UVB+), it increased significantly by 74.93% in the nNIR irradiation group and by 70.89% in the White irradiation group (*p* < 0.05). The collagen production increased by 2.40% in the nNIR irradiation group compared to that in the White irradiation group (Fig. 4).

To determine the inhibitory effect of nNIR on collagenase activity, we examined the mRNA expression of *Mmp9* and *Mmp13* in UV-induced photoaging mouse skin after nNIR or White irradiation. The relative *Mmp9* mRNA expression increased by 2.62-fold in the negative control group (UVB+) compared to that of the control group (UVB-). *Mmp9* mRNA expression was reduced by 0.92-fold in the nNIR irradiated group, and the collagenase mRNA expression level was reduced by 0.16-fold in the White irradiated group (Fig. 4). The relative *Mmp13* mRNA expression increased by 4.7-fold in the negative control group (UVB+) compared to that of the control group (UVB-). *Mmp9* mRNA expression was reduced by 0.50-fold in the nNIR irradiated group, and collagenase mRNA expression was reduced by 0.42-fold in the White irradiated group.

In photoaging mouse skin, MMP-13 protein expression was increased by 2.79-fold in the negative control group (UVB+) compared to that in the control group (UVB-) (Fig. 4). Consistent with the PCR results, MMP-13 protein expression decreased by 1.31-fold after nNIR irradiation compared to that in the negative control group. MMP-13 expression in the White-irradiated group was reduced by 1.48-fold (Fig. 4).

### Hair growth-promoting effect of nNIR in in vivo mouse model

**Fig. 5:**
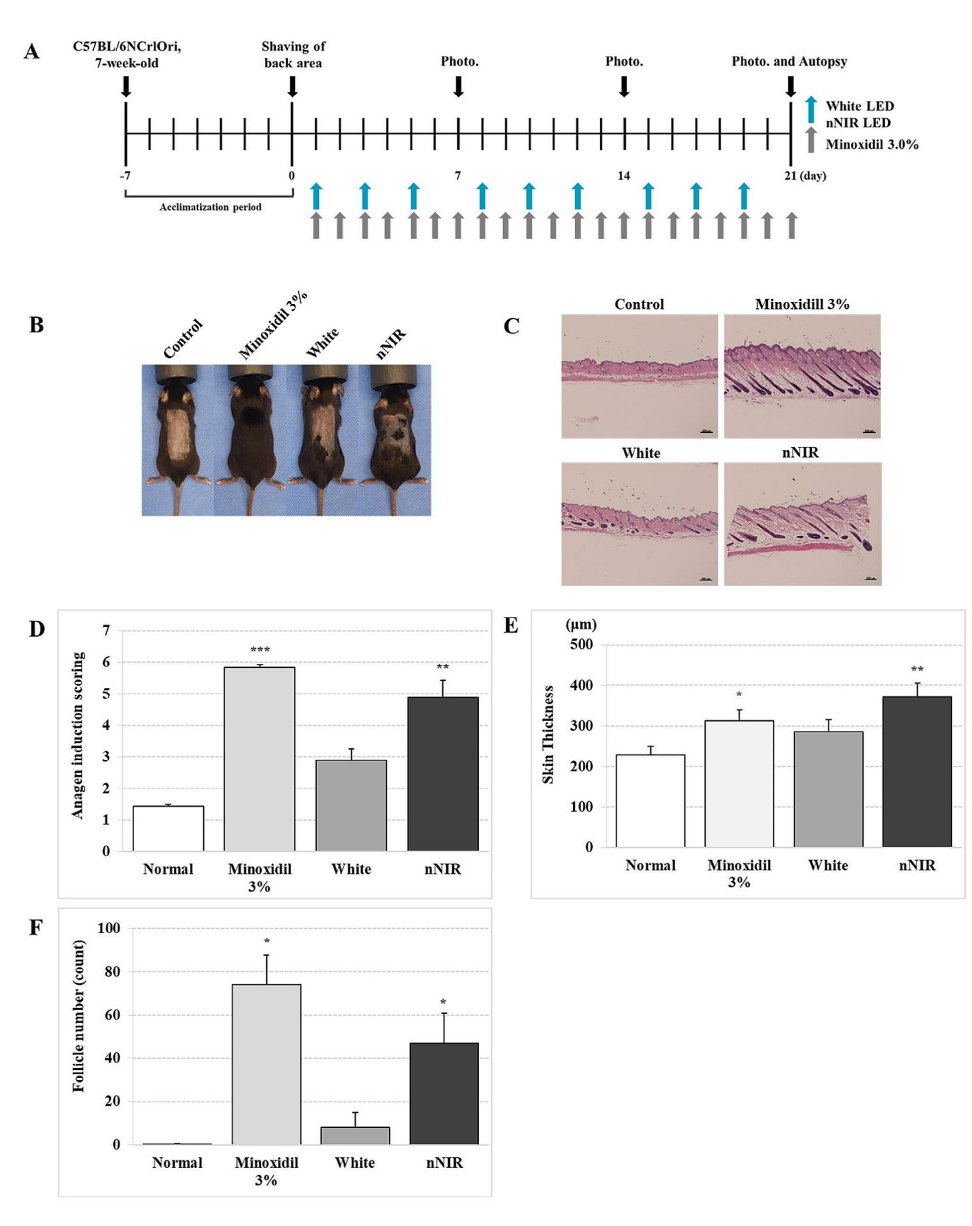
Effects of nNIR on anagen induction in 7-week-old C57BL/6 mice. (**A**) Schematic presentation of experimental timeline for hair growth model. (**B**) Gross image of hair regrowth in C57BL/6 mice with either Control, Minoxidil 3%, White and nNIR. (**C**) Hematoxylin and eosin (H&E) stain of mice dorsal skin. (**D**) Histological analysis of hair cycle score in mice. At least 10 hair follicles in the mice were evaluated in each mice group. (**E**) Histological analysis of full mice skin thickness. (**F**) Number of hair follicle per 1 mm section of back skin. Data are mean ± SEM. *N* = 5 mice per group. *P < 0.05, **P < 0.01, ***P < 0.001

To determine whether NIR LED can enhance hair growth in vivo, we irradiated NIR LED and White on the shaved back of the telogen phase in C57BL/6NCrlOri mice. Minoxidil (3%) was topically applied as a positive control [33]. We observed a color change from pink to gray skin, or the hair coat was larger on day 21 for the nNIR irradiated group compared with that of the control group. For the hair growth analysis, we used a well-defined assay to measure the induction of hair growth (Fig. 5).

Hair follicle anagen induction scores were assessed through histological analysis of dorsal skin, and when the anagen induction score was analyzed based on 100% of the control group, no light or substance was applied, the anagen induction score of the nNIR was 341.9%, and the White irradiated group showed an increase of 202.10%. NIR LED irradiation increased anagen induction by 1.69-fold more than White irradiation. As a result of skin thickness analysis, when the control group was analyzed based on 100%, nNIR irradiation increased skin thickness by 162.43%, whereas White irradiation increased it by 124.73%. In comparison between the LED irradiated groups, the nNIR irradiation increased skin thickness by 1.30-fold more than White irradiation (Fig. 5).

In the evaluation of the number of hair follicles, nNIR irradiation remarkably increased the number of follicles by 235-fold and White irradiation by 4,0-fold compared to the control group. Compared to the White irradiated group, the nNIR irradiated group showed an increase in the number of hair follicles by approximately 5.88-fold (Fig. 5).

Through visual and histological evaluation of in vivo mice, we suggest that nNIR irradiation and White stimulation stimulate hair growth in vivo. nNIR is more effective in improving anagen induction, skin thickness, and number of hair follicles than White irradiation. Thus, nNIR can promote hair growth more than White (Fig. 5).

In summary, this study developed an nNIR device with a wider wavelength than the conventional LED wavelength band and evaluated its anti-aging and hair growth capabilities in vitro and in vivo by comparing it with White.

## Discussion

With the spread of LEDs since 2010, indoor lighting has changed to LED replacing off-the-shelf lighting, such as fluorescent lights and incandescent lights. Unlike the wavelength of solar light (generated at high temperatures) consisting of 6% UV, 47% visible, and 53% NIR, LED is a technology that converts electrical to light energy and consists of narrow visible light of approximately 300 nm ranging from 400 nm to 700 nm [34]. Humans have adapted to solar light for at least hundreds of thousands of years; however, indoor activity began to increase in the late 1800s with the invention of incandescent lamps. Unlike in the past, modern people spend 90% of their daily lives indoors; therefore, they are greatly affected by artificial lighting. Moreover, the biological impact of light on humans is affected by the efficiency of lighting.

For years, scientists have studied the process by which solar light (UVB and UVA) ages the skin, causing wrinkles and discoloration. Recently, we started discussing the effects of visible light on the skin, and recently, researchers have aimed to better understand how visible light and LED affect the skin. LED has been in use since the 1960s; however, its use in skin treatment has only recently come into practice [11]. Different wavelengths of the visible spectrum correspond to different colors of LED and have different biological effects depending on the depth of penetration into the skin at different depths [35].

In this study, the first objective was to verify the effectiveness of LED sources that broadly cover Karu’s action spectrum. The second objective was to confirm whether Karu’s action spectrum functions as intended. Lastly, experiments were conducted to assess the potential harmful effects of White sources that encompass both blue and red wavelengths, with a particular focus on evaluating the high blue peak similar to that of a standalone blue LED source.

In Karu’s action spectrum, it is observed that different wavelengths of light exhibit relative effects on specific biological responses, similar to the absorption spectrum of transition metals Cu and Fe within the mitochondrial complex protein cytochrome c oxidase [36]. In the literature on action spectra, the generalized action spectrum reveals variations in wavelength and intensity depending on the oxidation and reduction states of CuA and CuB [37]. Thus, the action spectrum for biological responses consists of one or more wavelengths, suggesting the advantage of using a spectrum that encompasses a wider spectral range.

In this study, we developed a new NIR LED (nNIR) that emits a spectrum covering the action spectrum range of 600–900 nm, which is significantly wider than the conventional narrow-bandwidth NIR LED chips (20–30 nm). We compared the effects of the nNIR with the commonly used 2 chip LED sources in terms of improvement in human skin cells, specifically in the context of LLLT and PBM. Furthermore, we conducted experiments to evaluate the skin rejuvenation and hair growth effects of the nNIR in comparison to White sources commonly used for indoor lighting, using in vivo mouse models of aging and hair loss.

We verified the effects of nNIR on cells and animals. Almost all indicators related to hair and skin confirmed the effect of nNIR, compared to that of existing White sources, on improving skin wrinkles and promoting hair growth, and animal experiments that reflect permeability by wavelength confirmed the effect more clearly than cell experiments. This can be understood because when mitochondria receive the NIR wavelength, the blue-green wavelength penetrates the skin within 1 mm, whereas the red-NIR wavelength can penetrate the skin up to 8 mm [21]. Furthermore, ATP synthesis increases substantially when mitochondria receive NIR wavelengths.

ATP binds to purinergic receptors and various skin cells and tissues. The effect of ATP varies according to the cell type and concentration, and is associated with inflammation, wound healing, and psoriatic skin cancer [38]. We observed that the amount of ATP released from dermal cells increased substantially after irradiation with an NIR light source compared with other cells. Therefore, it is likely that ATP in dermal cells affects the surrounding cells. In addition, even in an in vivo environment, when irradiated with a light source, ATP can be released from skin cells, and the released ATP can have a positive effect on keratinocytes or surrounding cells [30].

In vitro, it was confirmed that the ATP production amount of nNIR for keratinocyte cells was higher than that of White irradiation, and it was observed that ATP synthesis was higher than that of White after nNIR irradiation even in the in vivo environment. Mitochondrial function and ROS generation are more specifically associated with epidermal homeostasis and hair follicle development and are known to aid in the regulation of stem cell differentiation [3, 39]. Thus, signaling through the mitochondria, such as ATP, is involved in skin structure and function. Therapeutic targeting of the mitochondria in the skin involves promoting ATP production or scavenging excess amounts of free radicals. For example, coQ10, an important ingredient in several anti-aging and regenerative creams, induces sufficient antioxidant supplementation [31]. CoQ10 also restores ATP production, prevents mitosis, and prevents oxidative stress in aging skin cells [40]. Nicotinamide is a precursor of NAD^+^ required for mitochondrial ATP production. The topical application of nicotinamide has anti-inflammatory effects against rosacea, acne, and hyperpigmentation [41]. It is also a popular additive for rejuvenating aged and sun-damaged skin [42]. In addition, NO production is known to be another important factor, along with mitochondria absorbing NIR energy, to regulate ATP generation and ROS [43]. This suggests that NO generation can have a positive effect on skin and hair by increasing bioenergy and promoting blood circulation [44].

The expression of the moisturizing factor *HAS3* mRNA [32] showed varying results depending on the dose and experimental conditions for each LED source, indicating the presence of optimal conditions concerning wavelength and dose. One clear finding is that all six LED sources, including 2chip, 680 nm, 760 nm, white, NIR white, and nNIR exhibited a significant increase ranging from 42 to 254% in skin moisturization compared to the control group. Recent studies have reported that irradiation of LLLT affects the expression of cytokines such as IL-1β in skin wound healing [14, 27]. Regarding the inflammatory cytokine markers, all LED sources demonstrated a similar level of inhibition of *IL-6*, *IL-8* and *TNF-α* mRNA compared to the inflammation-induced control group. However, the 680 nm and 760 nm chips showed data indicating that they were unable to suppress *IL-6* and *TNF-α* mRNA under the conditions of 4 h of exposure at 86.4 J/cm2.

Our results showed that White, commonly used in daily life, had some effect on skin improvement. Whites are known to be effective for the skin and are used as actual clinical equipment [23]. Nevertheless, the nNIR source developed by us increased ATP synthesis, collagen synthesis, and collagenase inhibitory activity more than the white light source and was strikingly effective for hair growth compared to the white light source in vivo.

Through application of the nNIR, which has a wider wavelength in the region that does not emit light in the currently used White, as a daily light, we can expect skin improvement and hair growth promoting effects. However, beyond cell and animal experiments, more research is needed on the power and dose that affect the human body, and additional mechanistic studies are needed.

In conclusion, this study examined the anti-aging and hair growth efficacy of nNIR not only in vitro but also in vivo, comparing it with White or 2chip LED. The results demonstrated that nNIR led to increased ATP synthesis and collagen synthesis, as well as decreased ROS levels. In a UV-induced photoaging mouse model, nNIR treatment resulted in increased ATP synthesis, decreased skin thickness, increased collagen synthesis, and reduced collagenase expression. Moreover, both NIR LED and White promoted hair growth, increased skin thickness, and stimulated follicle proliferation compared to the control group. Notably, the nNIR showed higher efficacy in improving ATP activity, enhancing collagen synthesis, suppressing collagenase expression, and promoting hair growth in vivo compared to conventional White. These findings suggest that the superior ATP synthesis activity of the NIR LED compared to White illumination contributes to these effects. Based on these results, we propose that the broader spectrum emitted by the near-infrared (nNIR) LED, in comparison to White and the conventional 2chip LED, induces more positive biological responses and ATP activity. This suggests that the observed effects can be attributed to the NIR LED’s ability to stimulate a wider range of biological processes. This research provides valuable insights for the future development of light-based therapies for skin rejuvenation and hair growth.

## Electronic supplementary material

Below is the link to the electronic supplementary material.

Supplementary material 1

## Data Availability

The data that support the findings of this study are available from the corresponding author upon reasonable request.
