# Supplementary material for: Enhancement of skin rejuvenation and hair growth through novel near-infrared light emitting diode (nNIR) lighting: in vitro and in vivo study
Source: Lasers Med Sci. 2024 Apr 17;39(1):104. doi: 10.1007/s10103-024-04044-9 (PMC11024053; doi:10.1007/s10103-024-04044-9)
Supplement: Supplementary file 1 — Supplementary material 1 [file 10103_2024_4044_MOESM1_ESM.docx]

**Supplementary data**

**
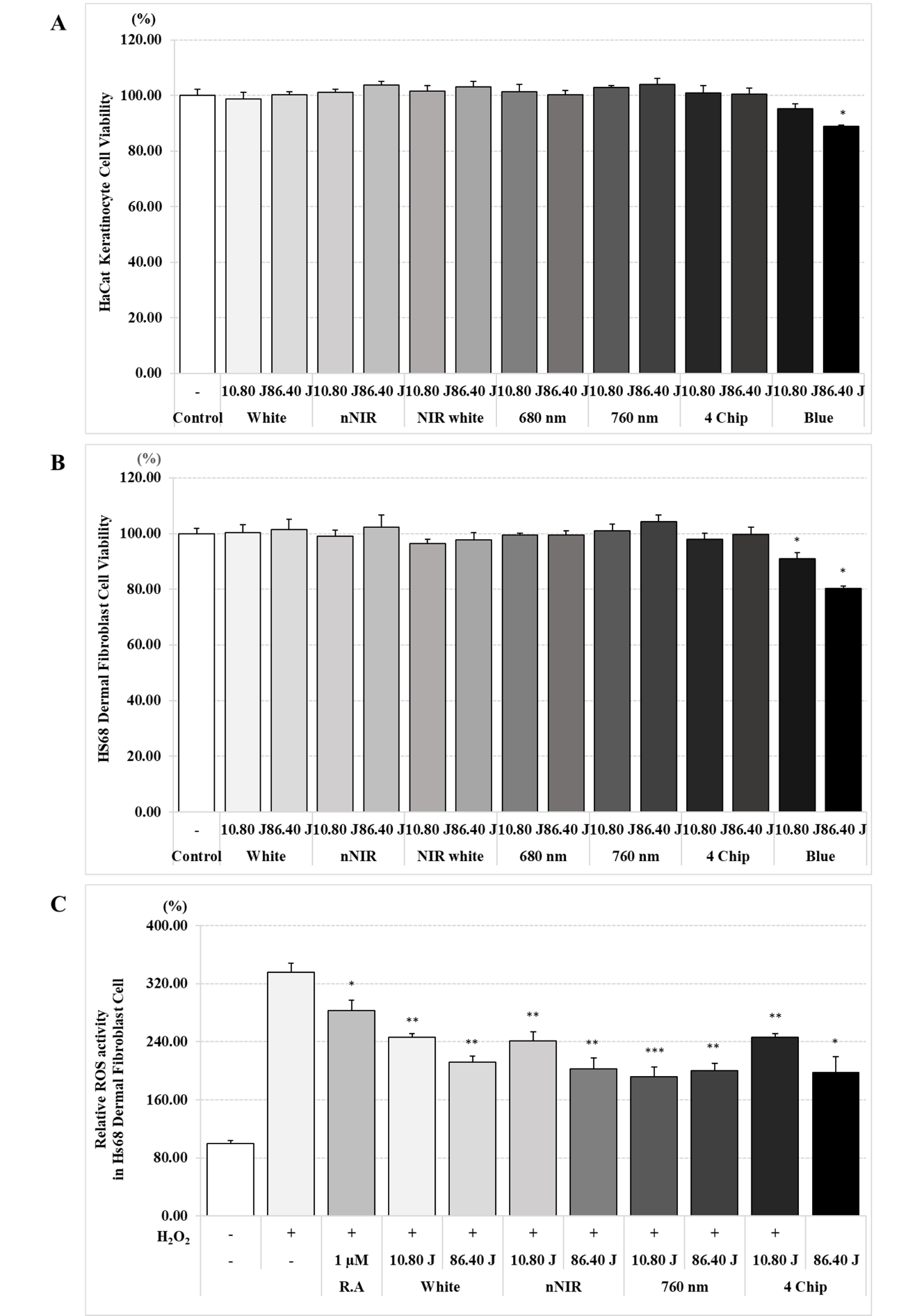
**

Sup Fig 1 Cytotoxicity and ROS activity by various light sources. A) Cytotoxicity of various light sources in Hs68 dermal fibroblast or HaCat keratinocyte cell B) Inhibition of ROS in Hs68 dermal fibroblasts. Data are mean ± SEM. N=3. **P* < 0.05, ***P* < 0.01


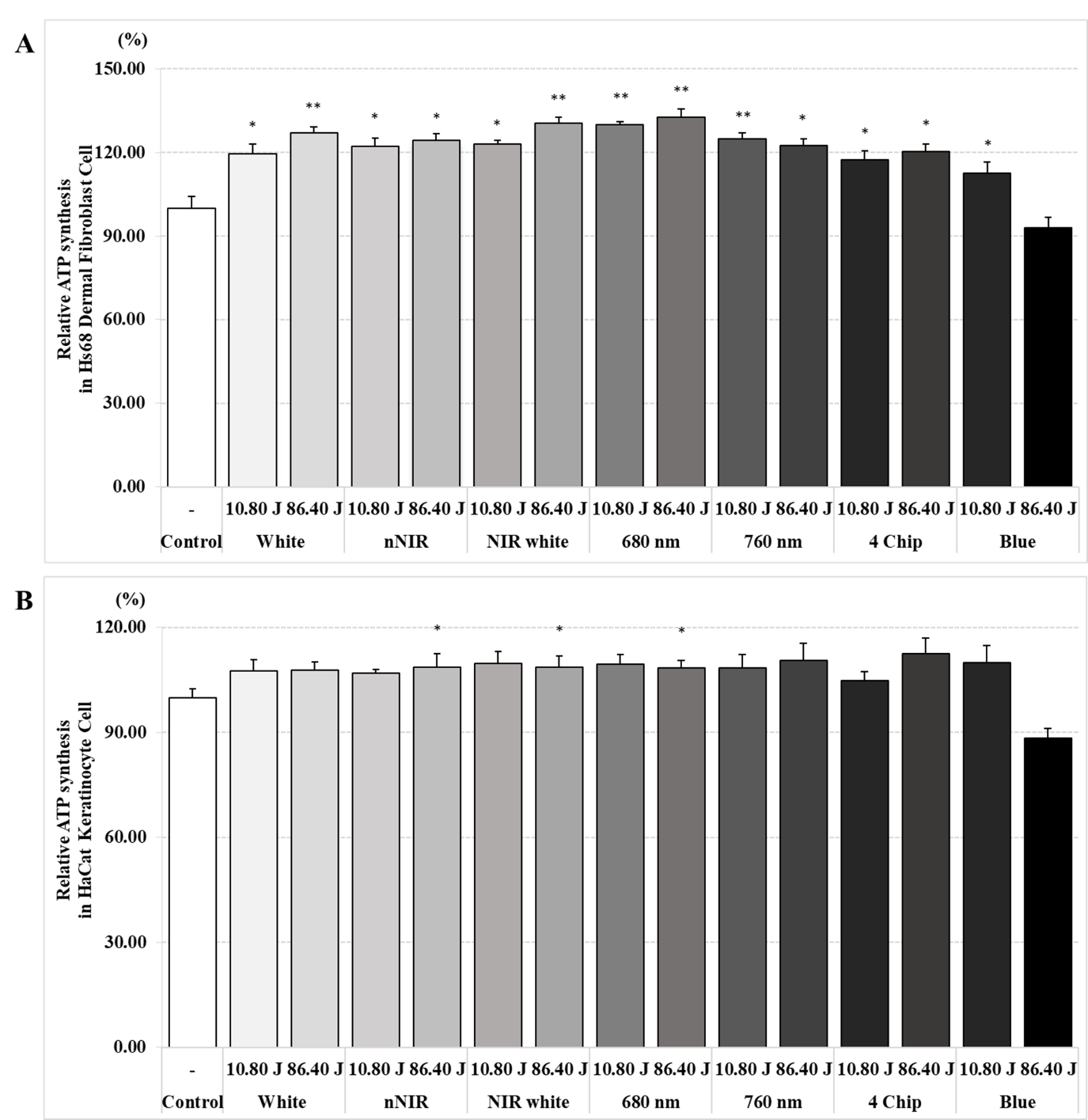


Sup Fig 2 Increase of ATP content by various LED sources in Hs68 dermal fibroblasts or HaCat keratinocyte. A) Relative comparative analysis of the amount of ATP synthesis in HS68 dermal fibroblast cells and B) in HaCat keratinocyte. Data are mean ± SEM. N=3. **P* < 0.05, ***P* < 0.01, ****P* < 0.001


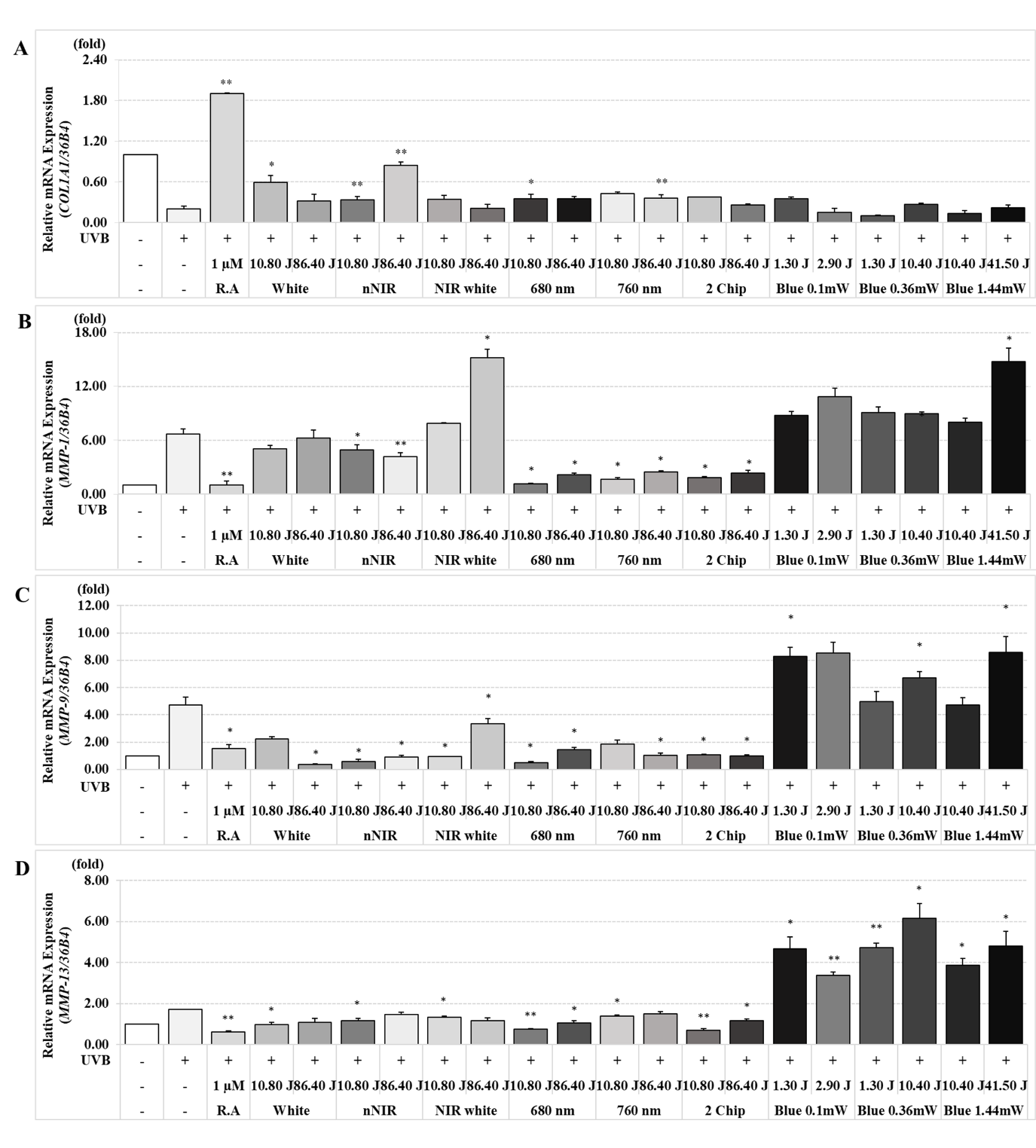


Sup Fig 3 Reduction of *Col1A1* mRNA or various collagenases by various light sources in Hs68 dermal fibroblasts. A) qRT-PCR analysis of *Col1A1* mRNA, B) qRT-PCR analysis of *MMP*1 mRNA C) qRT-PCR analysis of *MMP9* mRNA and D) qRT-PCR analysis of *MMP13* mRNA in HS68 dermal fibroblast cell. Data are mean ± SEM. N=3. **P* < 0.05, ***P* < 0.01


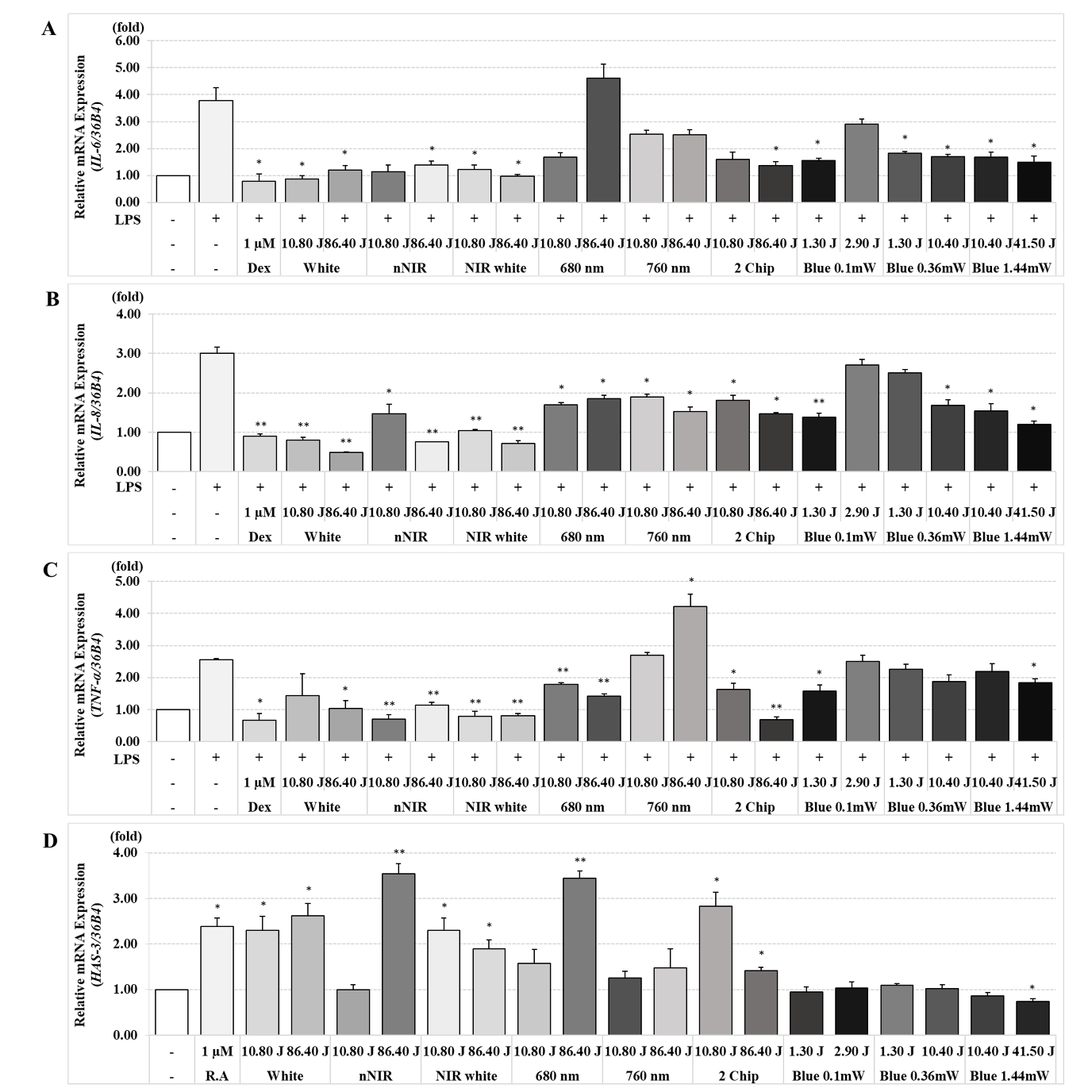


Sup Fig 4 Reduction of inflammatory factors mRNA and increased of *HAS3* mRNA by various light sources in HaCat keratinocyte. A) qRT-PCR analysis of *IL-6* mRNA, B) *IL-8* mRNA C) *TNF-α* mRNA and D) *HAS-3* mRNA in HaCaT keratinocyte cell. Data are mean ± SEM. N=3. **P* < 0.05, ***P* < 0.01, ****P* < 0.001
